# The modified Gingyo-san, a Chinese herbal medicine, has direct antibacterial effects against respiratory pathogens

**DOI:** 10.1186/s12906-016-1431-3

**Published:** 2016-11-14

**Authors:** Tetsuya Yamada, Takeaki Wajima, Hidemasa Nakaminami, Kaho Kobayashi, Hideaki Ikoshi, Norihisa Noguchi

**Affiliations:** 1 Department of Traditional Chinese Medicine, School of Pharmacy, Tokyo University of Pharmacy and Life Sciences, Tokyo, Japan; 2 Department of Microbiology, School of Pharmacy, Tokyo University of Pharmacy and Life Sciences, 1432-1 Horinouchi, Hachiouji, Tokyo 192-0392 Japan

**Keywords:** Modified Gingyo-san, Respiratory pathogen, Chinese herbal medicine

## Abstract

**Background:**

Modified Gingyo-san (MGS) is empirically used to treat various respiratory infections. MGS has been reported to have antiinflammatory and antiviral activities; however, it is not known if it has an antibacterial activity. Therefore, in this study, we aimed to investigate the antimicrobial activity of MGS against respiratory pathogens.

**Methods:**

MGS, which is sold as an over-the-counter drug in Japan, was used for the study. Antimicrobial activity was evaluated using the disk diffusion method. Growth inhibitory activity was evaluated by measuring colony-forming units of the pathogens in the presence of MGS.

**Results:**

MGS inhibited the growth of *Bacillus subtilis*, *Streptococcus pneumoniae*, and *Streptococcus pyogenes*, which are gram-positive bacteria. Although the growth of most gram-negative bacteria was not inhibited by MGS, interestingly, the growth of *Haemophilus influenzae* was inhibited. MGS did not show any activity against *Candida albicans* or bacteriophage φX174.

**Conclusions:**

In addition to the antiinflammatory and antiviral activities of MGS, which have already been reported, the data obtained from this study indicates that MGS has an antibacterial activity.

## Background

Respiratory infections are mainly caused by viruses or bacteria. Particularly, *Streptococcus pneumoniae*, *Haemophilus influenzae*, and *Streptococcus pyogenes* are the major causative bacteria of respiratory infections. In several cases of respiratory infections, the aforementioned bacteria cause severe invasive infections [1–3]. Furthermore, these bacteria are developing resistance to the currently used antimicrobial agents, which can result in various clinical concerns [4]. This indicates the urgent need for developing novel antimicrobial agents.

Until recently, the use of traditional medicines was focused mainly in alternative medicine. In traditional Chinese medicine, herbal medicines are used for the treatment of various respiratory infections. However, the use of such medicines is based on experience and is not supported by basic scientific evidence. These medicines have, however, been applied in many clinical settings. In recent years, many researchers have made efforts to establish basic scientific evidences for the use of several herbal medicines used in traditional Chinese medicine because of evidence-based treatment. For example, it has been reported that Sho-sei-ryu-to and Ma-o-to have antiviral activities against the influenza virus [5–7]. Gingyo-san (GS) has also been reported to have antiviral activity against influenza virus and an immunomodulating activity [8–11]. Modified Gingyo-san (MGS) and GS are sold as over-the-counter (OTC) drugs. MGS is used to treat sore throats, coughs, and headaches. GS and MGS are composed of the crude drugs shown in Table 1. The individual components of MGS have been studied by several researchers. The roots of glycyrrhiza and platycodon, which are included in MGS, have been reported to have antiinflammatory and antitussive activities [12, 13]. In addition, schizonepeta spike and forsythia fruit have antibacterial activities against *Propionibacterium acnes* and thus, they are used for treating acne [14–16].

**Table 1:** Formulations of modified Gingyo-san and Gingyo-san

Content	Modified Gingyo-san (MGS)^a^Amount (g)^b^	Gingyo-san (GS) Amount (g)^b^
Lonicerae Flos	4.26	30.0
Forsythiae Fructus	4.26	30.0
Glycyrrhizae Radix	2.56	15.0
Platycodi Radix	2.56	18.0
Menthae Herba	2.56	18.0
Arctii Fructus	2.14	18.0
Schizonepetae Spica	1.7	12.0
Fermented soybean	2.14	12.0
Lophatherum Herba	1.7	15.0
Antelope Horns	0.13	-

^a^extract

^b^daily dose

Therefore, MGS has a potential use in the treatment of various symptoms of infectious diseases. In this study, we evaluated the direct effects of MGS on pathogenic bacteria to establish a basic evidence of its antibacterial activity.

## Methods

### Microbial strains and culture conditions

The microbial strains used in this study are listed in Table 2. All the microbes, except the streptococci and *H. influenzae*, were cultured using Mueller-Hinton broth or agar (Oxoid Ltd., Hampshire, UK). The streptococci and *H. influenzae* were cultured using blood agar or Todd-Hewitt broth (Oxoid Ltd.) and chocolate agar or brain heart infusion broth (Oxoid Ltd.) supplemented with 15 μg/mL of nicotinamide adenine dinucleotide solution and 15 μg/mL of hemin solution, respectively.

**Table 2:** Bacterial, fungal and viral strains used in this study

Microorganism	Description
Gram-positive bacteria	
*Staphylococcus aureus* JCM2874	Quality control strain for susceptibility test, Methicillin-susceptible
*Staphylococcus aureus* N315	Methicillin-resistant *S. aureus*
*Streptococcus pneumoniae* ATCC49619	Quality control strain for susceptibility test, penicillin-susceptible
*Streptococcus pneumoniae* 19F	Clinical isolate, penicillin-resistant *S. pneumoniae*, serotype 19F
*Streptococcus pyogenes* JCM5674	Type strain
*Enterococcus faecalis* ATCC29212	Quality control strain for susceptibility test
*Bacillus subtilis* ATCC6633	Control strain for various assays
Gram-negative bacteria	
*Escherichia coli* ATCC25922	Quality control strain for susceptibility test
*Escherichia coli* C	Host of bacteriophage φX174
*Pseudomonas aeruginosa* ATCC27853	Quality control strain for susceptibility test
*Haemophilus influenzae* ATCC49247	Quality control strain for susceptibility test
*Serratia marcescens* ATCC13880	Type strain
Fungi	
*Candida albicans* ATCC10231	Quality control strain for various assays
Bacteriophage	
φX174	Virulent phage for *E. coli* C

### Disk diffusion method

MGS was obtained from ISKRA Industry (Tokyo, Japan). The disk diffusion antimicrobial test was conducted as follows. Briefly, 8-mm paper disks were impregnated with MGS suspension to obtain 8 mg of MGS/disk. The bacteria were suspended in 0.75% agar containing Mueller-Hinton broth and poured into suitable petri dishes. The disks were placed on the set agar and the plates were incubated at 35 °C overnight, after which the zones of growth inhibition were measured.

### Evaluation of growth inhibitory activity

A single colony of bacteria was inoculated into the appropriate broth medium and incubated at 37 °C overnight. The culture was diluted with the broth (1:100), with or without MGS, and incubated at 37 °C with shaking. A 100-μL of the culture was sampled at 0, 1, 2, 4, and 6 h after incubation and diluted with phosphate-buffered saline (PBS). Serial dilutions were then plated on the appropriate agar plates and incubated at 37 °C overnight. Afterwards, the numbers of grown colonies were counted. All the experiments were performed at least twice on independent days. It was also confirmed that all the experiments showed similar results.

### Plaque assay

MGS was mixed with bacteriophage φX174. The mixtures were diluted with PBS and further mixed with *Escherichia coli* C and 0.75% agar. The mixtures were then poured onto nutrient agar and incubated at 37 °C for 12 h, after which the plaques were counted.

### Cell proliferation assay

Cell proliferation activities with or without MGS were performed using CellTiter 96 Aqueous One Solution Cell Proliferation Assay Kit (Promega, Madison, MI, USA). Monolayer human lung epithelial cell lines (A549 cells) were grown using Eagle’s minimal essential medium, to which 10% fetal calf serum had been added, in 96-well plates. Aliquots of MGS were then added and the plates were incubated at 37 °C for 3 h under 5% CO_2_. After incubation, the numbers of living cells were determined using the kit according to the manufacturer’s instructions [17].

### Statistical analysis

We assessed statistical significance of differences for growth in the presence or absence of MGS. We performed Student’s and Welch’s *t*-tests using JMP software (SAS Institute Inc., NC, USA). *P* < 0.05 were judged as significant difference.

## Results and discussion

### Antimicrobial activity of MGS

The disk diffusion method is used as a screening test for the antimicrobial activities of drugs　and natural products [18, 19]. The susceptibility disk method was therefore used to determine whether MGS has antimicrobial activity (Table 3). Zones of growth inhibition were obtained in the experiments involving *S. pneumoniae* and *S. pyogenes* but not in those involving the other pathogens. This data indicates that MGS has a direct antibacterial activity against *S. pneumoniae* and *S. pyogenes*.

**Table 3:** Inhibitory zone of modified Gingyo-san containing paper disk^a^

Strain	Inhibitory zone (mm)^b^
*Staphylococcus aureus* JCM2874	-
*Staphylococcus aureus* N315	-
*Streptococcus pneumoniae* ATCC49619	10.0
*Streptococcus pneumoniae* 19F	10.0
*Streptococcus pyogenes* JCM5674	8.5
*Enterococcus faecalis* ATCC29212	-
*Bacillus subtilis* ATCC6633	-
*Escherichia coli* ATCC25922	-
*Pseudomonas aeruginosa* ATCC27853	-
*Haemophilus influenzae* ATCC49247	-

^a^8 mg/disk

^b^- , inhibitory zone not appeared

### Growth inhibitory activity of MGS against respiratory pathogens

**Fig. 1:**
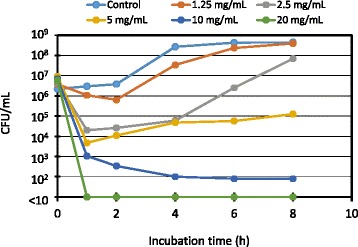
Antibacterial effect of modified Gingyo-san against *Bacillus subtilis* ATCC6633. This experiment was performed twice on independent occasions and similar results were obtained. The data shown is representative of the results obtained

The susceptibility disk test is suitable for screening the antimicrobial activities of drugs; however, not all drugs that have antimicrobial activities produce zones of growth inhibition, which may be due to the chemical properties of the drugs [20]. Therefore, to validate the growth inhibitory effect of MGS, bacterial colony-forming units (CFUs) in cultures were monitored over time in the presence or absence of MGS. The number of CFUs of *B. subtilis* ATCC6633, which is normally used for testing the antibacterial activity of drugs, decreased in a dose-dependent manner after the addition of MGS to the culture medium (Fig. 1). MGS is usually administered with approximately 100 mL of water; thus, after administration, the concentration of MGS in the oral cavity reaches approximately 24 mg/mL. Since MGS was used at a concentration equivalent to its usually administered dose, the data obtained indicates that MGS has a direct antibacterial effect at its normal dose.

**Fig. 2:**
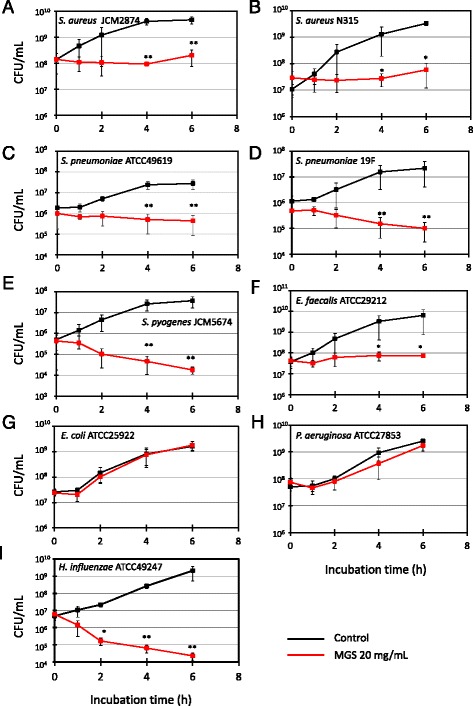
Antibacterial effects of modified Gingyo-san against several bacterial strains. **a** *S. aureus* JCM2874; **b** *S. aureus* N315 (MRSA); **c** *S. pneumoniae* ATCC49619; **d** *S. pneumoniae* 19F; **e** *S. pyogenes* JCM5674; **f** *E. faecalis* ATCC29212; **g** *E. coli* ATCC25922; **h** *P. aeruginosa* ATCC27853; and **i** *H. influenzae* ATCC49247. Each experiment was performed three times on independent occasions and similar results were obtained. The *P* value was calculated by Welch’s *t*-test. ** *P* < 0.01, *P < 0.05

The antimicrobial activity of MGS was analyzed at a concentration was 20 mg/mL. The growth of *S. aureus*, *S. pneumoniae*, *S. pyogenes*, and *Enterococcus faecalis* was significantly inhibited by the addition of MGS to the respective culture media (Fig. 2). MGS also inhibited the growth of antimicrobial-resistant strains such as methicillin-resistant *S. aureus* (MRSA) and penicillin-resistant *S. pneumoniae* (PRSP) (Fig. 2). Therefore, these results indicate that MGS has growth inhibitory effects against gram-positive bacteria. However, the growth of *E. coli* and *Pseudomonas aeruginosa* was not inhibited (Fig. 2). In addition, MGS did not affect other gram-negative bacteria such as *Acinetobacter baumannii*, *Serratia marcescens*, and *Klebsiella pneumoniae* (data not shown).

However, interestingly, the growth of *H. influenzae*, which is a major causative pathogen of respiratory infections, was significantly inhibited in spite of it being a gram-negative bacterium (Fig. 2). It has been reported that among gram-negative bacteria, *H. influenzae* has a different cell surface structure [21]. In addition, the chromosomal efflux pumps of *H. influenzae* are fewer than those of other gram-negative bacteria [22]. The aforementioned factors might therefore be related to the observed bactericidal effect of MGS on *H. influenzae* in this study. These findings indicate that MGS can comprehensively inhibit the growth of respiratory bacterial pathogens. Moreover, MGS showed inhibitory effects against MRSA and PRSP, which suggests that MGS could be a very useful antibiotic for the treatment of respiratory infections.

### Effects of MGS on *Candida albicans* and bacteriophage φX174

**Fig. 3:**
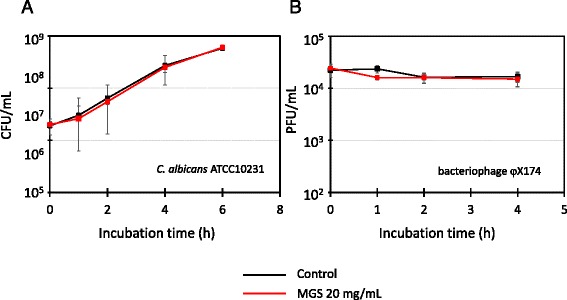
Antifungal and antibacteriophage effects of modified Gingyo-san. **a** *C. albicans* ATCC10231 and **b** bacteriophage φX174. Each experiment was performed 3 times on independent occasions and similar results were obtained

**Fig. 4:**
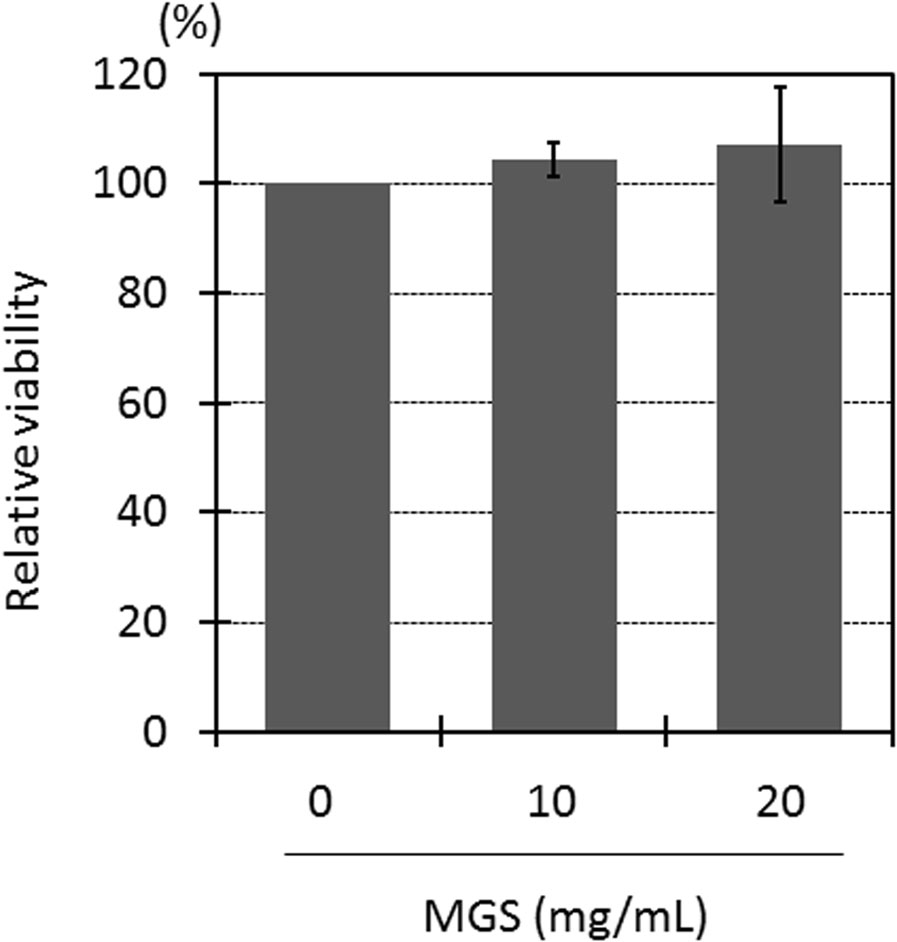
Effect of modified Gingyo-san on a eukaryotic cell. Proliferation activity was evaluated. Each experiment was performed 3 times on independent occasions. The *P* value was calculated by student’s *t*-test

The inhibitory effects of MGS on eukaryotes and viruses were analyzed using *Candida albicans* and bacteriophage φX174 as the eukaryotic and viral models, respectively (Fig. 3). The data obtained indicated that MGS does not inhibit the growth of *C. albicans* or bacteriophage φX174. In addition, MGS did not show any antiproliferative effects against the A549 cells (Fig. 4). These findings indicate that MGS might not have any direct effect on eukaryotes or viruses.

## Conclusions

Our study indicated that MGS has a direct antibacterial effect against respiratory bacterial pathogens. These findings together with those from previous reports show that, GS has in vivo antiviral activity against the influenza virus and that MGS could be a useful antibacterial agent. Therefore, MGS may be effective for use as a pastille and a gargle. Moreover, future studies need be conducted to clarify whether the metabolites of MGS as well have effects on bacteria.

## Abbreviations

GS: Gingyo-san
MGS: The modified Gingyo-san
